# Effects of Transcranial Direct Current Stimulation (tDCS) and Approach Bias Modification (ABM) training on food cravings in people taking antipsychotic medication

**DOI:** 10.1186/s13063-020-4112-y

**Published:** 2020-03-06

**Authors:** Luiza Grycuk, Gemma Gordon, Fiona Gaughran, Iain C. Campbell, Ulrike Schmidt

**Affiliations:** 1 Section of Eating Disorders, Department of Psychological Medicine, Institute of Psychiatry, Psychology & Neuroscience, King’s College London, London, SE5 8AF UK; 2 Department of Psychosis Studies, Institute of Psychiatry, Psychology & Neuroscience, King’s College London, London, SE5 8AF UK; 3 South London and Maudsley NHS Foundation Trust, Maudsley Hospital, London, SE5 8AZ UK

**Keywords:** Schizophrenia, Weight gain, Transcranial direct current stimulation, Approach bias modification training

## Abstract

**Background:**

Antipsychotic drug-induced weight gain puts individuals with schizophrenia at increased cardiometabolic risk. As a potential intervention for this problem, we describe the theoretical background and a protocol for a feasibility randomised controlled trial (RCT) of approach bias modification (ABM) training combined with real versus sham (placebo) transcranial direct current stimulation (tDCS). The primary aim of this trial is to obtain information that will guide decision making and protocol development in relation to a future large-scale RCT of ABM and tDCS in this group of participants. Second, the study will assess the preliminary efficacy of ABM + tDCS in reducing food cravings in people who take antipsychotic medication.

**Methods:**

Thirty adults with a DSM-V diagnosis of schizophrenia or schizoaffective disorder treated with anti-psychotic medication will be randomly allocated to receive five sessions that will combine ABM and real or sham tDCS, in a parallel group design. In this feasibility study, a broad range of outcome variables will be examined. Measures will include food craving, psychopathology (e.g. symptoms of schizophrenia and depression), neuropsychological processes (such as attentional bias and impulsiveness), and the tolerability and acceptability of tDCS. The feasibility of conducting a large-scale RCT of ABM + tDCS and appropriateness of tDCS as a treatment for antipsychotic drug-induced weight gain will be evaluated by assessment of recruitment and retention rates, acceptability of random allocation, blinding success (allocation concealment), completion of treatment sessions and research assessments (baseline, post-treatment and follow-up).

**Discussion:**

The effect sizes generated and other findings from this trial will inform a future large-scale RCT with respect to decisions on primary outcome measures and other aspects of protocol development. In addition, results from this study will provide a preliminary indication of the efficacy of ABM + tDCS treatment for antipsychotic drug-induced weight gain.

**Trial registration:**

ISRCTN Registry, ISRCTN13280178. Registered on 16 October 2018.

## Background

Individuals taking antipsychotic medication show increased food craving, caloric intake and weight gain which puts them at elevated risk for obesity-related conditions, e.g. type 2 diabetes and cardiovascular disease [1]. People with schizophrenia have a higher mortality rate than the general population, mainly due to physical illnesses [2]. Reducing the weight-related side effects of antipsychotic medication has the potential to improve health outcomes for this population.

Antipsychotic drug-induced weight gain is well documented. A meta-analysis of 81 studies reported that, after 10 weeks of treatment, there was a mean weight increase of 4.45 kg in patients receiving clozapine and 4.15 kg for those receiving olanzapine [3]. Fountaine et al. [4] showed that olanzapine treatment resulted in an estimated 345 kcal/day (18%) excess energy intake in 30 healthy male volunteers and 2.65 kg increased body weight (over 15 days). Another study showed that four weeks of treatment with olanzapine was associated with an estimated increase of energy intake of 598 kcal/day (28%) in 10 male adolescents [5]. These reports are broadly consistent with another review which concluded that patients with schizophrenia are more likely than matched controls to consume a diet poor in fibre and fruit and rich in saturated fat [6].

Treatments for antipsychotic drug-induced weight gain include medication and behavioural interventions such as nutritional advice, cognitive behavioural therapy and exercise. Pharmacological interventions (e.g. fenfluramine, sibutramine, reboxetine, metformin, topiramate) are not very effective and can have significant side effects [7], whereas research on behavioural interventions has produced mixed results. In a five-year naturalistic study of 82 outpatients newly started on clozapine, weight gain occurred despite active weight loss programmes involving diet and exercise [8]. A meta-analysis of 20 trials of exercise interventions reported no significant effect on body mass index (BMI) [9], whereas a review of 13 studies investigating behavioural interventions reported a weight loss of 3.15% of initial weight, well below the 5%–10% threshold considered sufficient to improve weight-related complications [10]. Another meta-analysis of 17 studies concluded that behavioural interventions prevented and/or reduced antipsychotic-associated weight gain (3.12 kg less weight gain); however, weight was significantly improved only in outpatient trials (*p* < 0.0001) but not in inpatient or mixed samples (*p* = 0.09–0.96) [7]. On the basis of these findings, there is a need for new treatments that target weight gain in people who take antipsychotic medication, especially those who may find it hard to engage in exercise or therapy.

Human and animal studies suggest that antipsychotic drugs stimulate appetite by interacting with dopamine (D2), serotonin (5HT2a & 5HT2c) and histamine (H1, H2) receptors [1]. Changes in peripheral hormones, e.g. leptin, ghrelin and adiponectin, have been reported to be involved [11]. Fat deposition may be facilitated by stress induced activation of the hypothalamic-pituitary-adrenal axis [12]. Genetic predisposition may also play a part, e.g. antipsychotic drug-induced weight gain is reported to be correlated with polymorphisms in the common promoter region for 5HT2c receptors [13] and polymorphisms near the melanocortin 4 receptor gene (MC4R4) [14].

As a result of altered appetite and increased susceptibility to hunger, people taking antipsychotic medication may develop disordered dietary behaviours [15]. Brömel et al. [16] showed that out of 12 patients started on clozapine, nine reported increased appetite and two developed binge-eating episodes. In another study of 74 patients on either olanzapine or clozapine, 37 screened positively for binge eating, with nine fulfilling criteria for binge-eating disorder and five for bulimia nervosa [17]. Additionally, patients who screened positively for binge eating showed higher BMIs and higher BMI gains during treatment. These results suggest that modifying food cravings and/or food consumption may affect antipsychotic drug-induced weight gain.

The observations described above are consistent with evidence from neuroimaging studies. A functional magnetic resonance imaging (fMRI) study of 25 individuals after one week of olanzapine treatment showed enhanced anticipatory and consummatory responses to food rewards and decreased responsivity to food consumption [18]. Another study of 25 individuals after 16 weeks of olanzapine treatment reported increased sensitivity to appetitive stimuli in insular cortices, amygdala and cerebellum, compared with controls [19]. There was also an increased response to appetite-related stimuli from baseline to post treatment, in the frontal cortex, fusiform gyrus, amygdala and insula.

Neural activity in certain brain areas can be enhanced or reduced by neuromodulation procedures, e.g. repetitive transcranial magnetic stimulation (rTMS) and transcranial direct current stimulation (tDCS). These non-invasive brain stimulation methods have demonstrated therapeutic potential in major depressive disorder [20], bipolar affective disorder [21], obsessive compulsive disorder, generalised anxiety disorder and substance use disorder [22]. They have also been trialled and well received in people with schizophrenia, helping to alleviate auditory verbal hallucinations and improving negative symptoms [22].

Brain stimulation may also be a promising tool for reducing food cravings [23] which could be used to treat antipsychotic-induced weight gain. The most common target of neuromodulation is the dorsolateral prefrontal cortex (dlPFC) which has been associated with control of eating via possible mechanisms of reward valuation, attention and inhibitory control [24]. For example, one session of high-frequency rTMS delivered to the left dlPFC lowered cue-induced food cravings in people with bulimic disorder [25, 26] and tDCS applied to the dlPFC reduced food cravings in healthy participants [27, 28] and the desire to eat in overweight and obese participants [29].

Another potential intervention for antipsychotic drug-induced weight gain is approach bias modification (ABM) training. ABM is a computer training aiming to modify implicit approach biases through teaching participants to avoid negative stimuli [30]. A review of 12 meta-analyses concluded that a course of ABM sessions can shift target biases in adults, with moderate effect sizes [31]. ABM can also be effective at re-training approach bias to appetitive cues such food and alcohol [32]. ABM significantly reduced approach tendencies and attention towards food cues in a sample of people who binge eat [33] and reduced eating disorders symptoms in a sample of people with either bulimia nervosa or binge-eating disorder [34].

TDCS has been proposed to modulate neural activity by changing the threshold for discharge of the stimulated neurons [35], i.e. it does not induce changes in neuronal firing in resting neuronal networks. Because tDCS does not alter resting networks, it has been proposed [36] that the potential therapeutic effects of tDCS are likely to be improved by pairing it with the behaviour (and associated changes in neuronal activity) that one is seeking to modify [36] (e.g. bias towards high-calorie foods). In this way, the effects of ABM may be enhanced by tDCS, i.e. it may increase neuroplasticity [37] and potentially aid learning aimed at avoiding high-calorie foods. In fact, this has been reported by Heeren et al. [38], who found that neuromodulation boosted the effects of cognitive training aimed at reducing cognitive bias and improving response inhibition. Den Uyl et al. [39] conducted four sessions of concurrent ABM and tDCS over seven days on alcohol-dependent inpatients. Although no enhanced effect of tDCS on ABM training was found, a reduced probability of relapse at the one-year follow-up was noted in the real tDCS group compared to sham. This indicates that combined tDCS and ABM can potentially have a stronger effect on reducing food cravings than either of the treatments alone.

In summary, research shows that dietary behaviours can be altered by neuromodulation methods as well as ABM training. To our knowledge, this will be the first time that both these interventions will be combined and applied in people taking antipsychotic medication. The proposed feasibility study is a randomised controlled trial (RCT) comparing ABM training combined with real (active) or sham (placebo) anodal tDCS to the right dlPFC in individuals with schizophrenia who take antipsychotic medication. We will assess recruitment, attendance, retention and follow-up rates that will inform the development of a large-scale RCT. Changes to food cravings and eating behaviours as well as other clinical outcomes (e.g. depression, anxiety, impulsiveness, schizophrenia symptoms) will be measured before and after the treatment intervention and at a two-week follow-up.

### Aims

The aims of the present study are to:

1. establish the feasibility of conducting a large-scale RCT of ABM + tDCS in people with schizophrenia who take antipsychotic medication: this will involve assessing safety (adverse events), recruitment, willingness to undergo random allocation to five sessions of ABM combined with real or sham tDCS, attendance and retention rates;
2. determine the best instruments for measuring outcomes in a future full trial by examining the quality, completeness and variability in the data;
3. estimate the treatment effect sizes and standard deviations for outcome measures to inform the sample size calculation for a large-scale RCT;
4. explore participants’ views on the acceptability, credibility, tolerability and experience of ABM + tDCS.

Based on neuromodulation studies conducted by our group [40, 41] and others [42–44] in people who experience food cravings, we predict that, compared to ABM + sham tDCS treatment, five sessions of ABM + real tDCS applied to the dlPFC will:

1. decrease approach bias towards food stimuli;
2. decrease state food craving after cue exposure;
3. decrease trait food craving from baseline to post assessment;
4. be considered by patients as an acceptable and useful treatment for antipsychotic drug-induced weight gain.

## Methods

### Design

**Fig. 1:**
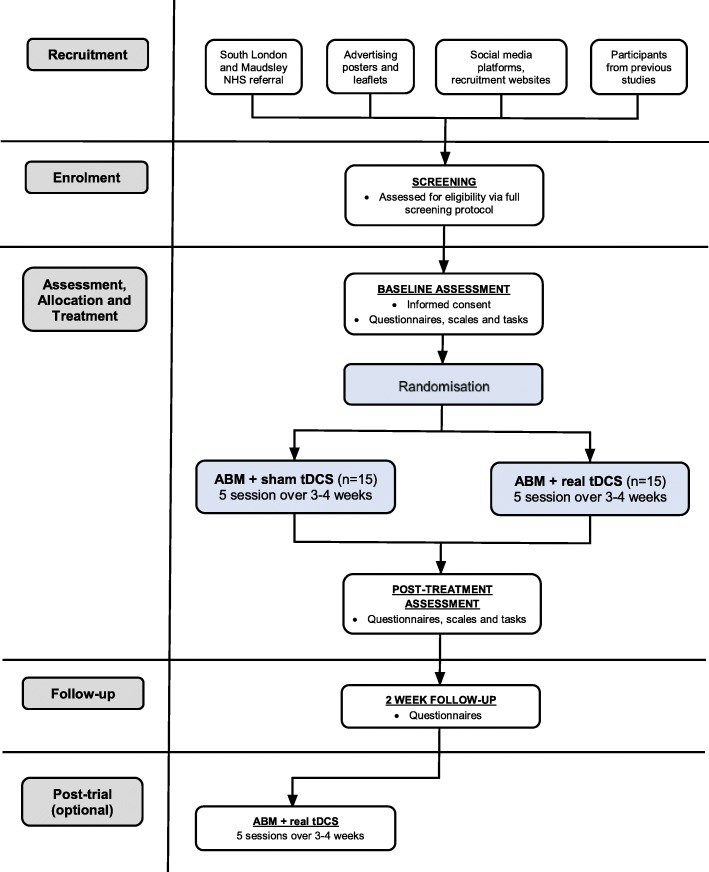
Schematic diagram of the study protocol

This is a parallel group, double-blind, two-arm RCT. Participants will be randomly allocated to receive five sessions of either ABM + real tDCS (treatment group) or ABM + sham tDCS (control group) over 3–4 weeks, delivered in addition to treatment as usual. Outcomes will be measured at baseline, after treatment and at the two-week follow-up. Participants in the control group will be offered the opportunity to receive ABM + real tDCS at the end of the study. The protocol is outlined in Fig. 1; Table 1 gives details of assessments and timepoints. Our study design will allow us to establish whether adding tDCS to the ABM is better than ABM alone. Specifically, this will be achieved by administering the Food Approach-Avoidance Task (F-AAT) and the Stimulus Response Compatibility Task (SRC) at baseline and after treatment to measure approach bias towards high-calorie foods in the two groups (real-tDCS and ABM vs sham-tDCS and ABM).

**Table 1:** Study schedule of enrolment, interventions and assessments

	Study period						
	Screen visit (all participants)	Baseline (all participants)	Training: ABM + real tDCS	Training: ABM + sham tDCS	Post-assessment (all participants)	Follow-up (all participants)	Study end (all participants)
Timepoint	–t1	0	t1	t1	t2	t3	t4
Participant information sheet, inclusion /exclusion criteria and tDCS safety screen	X						
Informed consent		X					
Demographic information		X			X		
Questionnaires		X			X		
Food-related tasks		X			X		
Approach bias assessment tasks		X			X		
Pre-ABM + tDCS measures: multiple VASs, blood pressure and pulse			X	X			
Anodal real tDCS to dlPFC			X				
Anodal sham tDCS to dlPFC				X			
Approach bias modification training			X	X			
Post ABM + tDCS measures: multiple VASs, blood pressure and pulse			X	X			
Tolerance, discomfort and side effects of tDCS			X	X			
Acceptability questionnaire					X		
Blinding assessment questionnaire					X		
Follow-up questionnaires						X	
Unblinding							X

*ABM* approach bias modification, *dlPFC* dorsolateral prefrontal cortex, *tDCS* transcranial direct current stimulation, *VAS* visual analogue scale

### Setting

The study will be conducted at the Institute of Psychiatry, Psychology and Neuroscience (IoPPN) and at inpatient and community services at the South London and Maudsley NHS Foundation Trust (SLaM).

### Ethical approval and trial registration

Ethical approval for the study was obtained from the Oxford B Research Ethics Committee (REC; reference no. 19/SW/0095). The study is registered on the International Standard Randomised Controlled Trial Number (ISRCTN) registry (registration no. ISRCTN13280178).

### Participants and recruitment

Participants will be recruited from inpatient and community services at the SLaM, through websites (such as IoPPN), through social media platforms (such as the Eating Disorders Unit’s official Twitter account) and through the Consent 4 Contact SLaM initiative [45]. Participants will be paid £90 for their time and effort.

### Inclusion criteria

Male or female participants will be included if they have a current DSM-V diagnosis of schizophrenia or schizoaffective disorder, are aged 18–65 years and have been on a stable dose of antipsychotic medication for at least six weeks before study enrolment.

### Exclusion criteria

Participants will be excluded if they: suffer from any significant/unstable co-morbid medical or psychiatric disorders (e.g. substance dependence); are on a dose of antidepressant medication that has not been stable for at least six weeks; are allergic to any of the foods used in the study; or cannot understand verbal or written English. A tDCS safety questionnaire will be administered and individuals will be excluded if they: have a history of epileptic seizures, stroke or brain injury; have any implanted metal devices in the head; suffer from frequent or severe headaches or dizziness; are pregnant.

### Sample size

As this is a feasibility study, no a priori sample calculation has been conducted. This study aims to provide effect sizes on which future large-scale studies can be based. Total sample sizes of 24–50 have been recommended for feasibility trials with a primary outcome measured on a continuous scale, mainly because estimates of the standard deviation for normally distributed variables tend to stabilise around this size [46, 47]. We have chosen a total sample size of 30 (which exceeds the lower end recommended for feasibility trials).

### Randomisation

Generation and implementation of the randomisation sequence will be conducted independently from the trial team by a King’s College researcher using Sealed Envelope, an online randomisation tool [48]. Once the baseline assessment has been conducted and the patient is recruited and has consented to the trial, he/she will be allocated to one of the two intervention arms in a ratio of 1:1. Group allocation will be communicated via phone, email or in a sealed non-transparent envelope to the appropriate member of the research team for each participant.

### Intervention

#### Study procedures

In both groups, participants will receive five sessions of ABM + real/sham tDCS over 3–4 weeks. ABM and tDCS will be delivered at the same time, i.e. participants will engage in the ABM while receiving brain stimulation. Each session will last approximately 40 min, including preparation time, 20 min of ABM + tDCS and questionnaire administration. The ABM will start 1 min after the start of the brain stimulation, to allow participants to get used to the brain stimulation. Thereafter, ABM will take place over 15 min and brain stimulation will then continue for a further 4 min. Throughout the study, participants will be able to access or continue treatment as usual as recommended by their treating team.

#### ABM training

The ABM programme is based on a modified version of the Food Approach /Avoidance Task (F-AAT). In the F-AAT task, participants are shown pictures of food and control (i.e. neutral household and office) items. They are required to pull (pictures grow bigger) or push (pictures grow smaller) a joystick in response to the outer frame of the picture (round vs rectangular), irrespective of the picture content. The ABM task adopts an implicit learning paradigm by presenting all food pictures in the ‘push’ (i.e. avoid) format. The study procedure for ABM administration is in accord with a protocol paper [49], with updated images of foods and non-edible objects from a food-pictures database [50].

#### tDCS

TDCS (both real and sham) will be delivered using a neuroConn® DC-STIMULATOR device at a constant current of 2 mA (with a 10-s fade in/out) using two 25 cm^2^ surface sponge electrodes soaked in a sterile saline solution (0.9% sodium chloride). The anode will be placed over the right dlPFC and the cathode over the left dlPFC. The stimulation site will correspond to the F3 location based on the International 10–20 system [51]. In the real tDCS group, current will be delivered for the whole duration of the stimulation (20 min). In the sham (placebo) tDCS group, current will automatically turn off after 30 s.

#### Safety

Study procedures and parameters are in accord with safety and application guidelines for tDCS [52]. Treatment will be delivered by personnel trained in tDCS administration. A case record form for each trial participant will be kept to monitor session attendance and any side effects or adverse events according to prespecified criteria. Any protocol violations will also be recorded there. To ensure safety, participants’ blood pressure and pulse will be monitored before and after each stimulation. TDCS is generally well-tolerated and is associated with relatively minor side effects. According to the review of 567 tDCS sessions, adverse events (and occurrence rates) included: tingling sensation (70.6%); moderate fatigue (35.3%); light itching sensation under the stimulation electrodes (30.4%); headache (11.8%); nausea (2.9%); and insomnia (0.98%) [53]. Another review of 209 tDCS studies found similar rates of adverse events in both real and sham stimulation groups [54]. In the event of mild side effects (e.g. a slight headache) participants will not be withdrawn but will be able to discontinue tDCS treatment if they wish. TDCS will be immediately halted if the participant experiences a more serious adverse event or if any other indicators of serious medical risk emerge. Treatment will only be restarted if it is deemed safe to continue by a medical professional. Standard King’s College London insurance and NHS indemnity arrangements will apply to this study.

### Procedure

A flowchart outlining study procedures is presented in Fig. 1. Table 1 presents the time schedule of enrolment, interventions and assessments, consistent with the figure provided in the SPIRIT Statement (2013) [55] recommendations for reporting protocols (see Additional file 1 for SPIRIT checklist).

#### Screening

Potential participants will be referred by their clinician or will self-refer. Researchers will screen participants for eligibility. Screening questionnaires include a tDCS safety screen and a short inclusion/exclusion study specific screen, including an assessment of medical and psychiatric history, and medication dosage and stability. In line with the CONSORT guidelines [56, 57], we will record the number and reasons for any participants we must exclude or any who decline consent or withdraw from the study.

#### Baseline assessment

Once eligibility has been confirmed, the participant’s written informed consent will be obtained by the researcher. Participants will be asked to complete a number of questionnaires and experimental procedures assessing eating behaviours and mood, as well as computer tasks that assess attention bias towards food cues. Once the baseline assessment is complete, participants will be randomly allocated to the treatment ABM + real tDCS or control ABM + sham tDCS groups.

#### Post-treatment assessment

Post-treatment assessment will take place after the last treatment session and include the same elements as the baseline assessment. Blinding success will be evaluated by asking participants and researchers to guess the treatment allocation.

#### Follow-up

Two weeks after post-treatment assessment, a follow-up session will be conducted. This short session will consist of questions regarding mood, food cravings and eating behaviours. Participants’ weight will be measured.

### Measures

#### Screening measures

A tDCS safety screen will be conducted to check for contraindications to tDCS.

#### Outcome measures

Since this is a feasibility study, a broad range of outcome measures are included to determine which are most sensitive to detecting a treatment effect. This will enable us to determine primary outcome(s) for a future large-scale RCT.

#### Clinical outcomes related to eating behaviours

1. Questionnaires including Eating Disorder Examination Questionnaire (EDE-Q) [58] and Food Cravings Questionnaire-Trait-Reduced (FCQ-T-r) [59] will be administered at baseline and after treatment. The FCQ-T-r will also be administered at a two-week follow-up.
2. Food tasks including the Food Challenge Task (FCT) [40] examining cue-induced food craving and the Taste Test measuring actual food consumption will be administered at baseline and after treatment. Within each session, visual analogue scales (VASs) regarding current experiences (level of hunger, feeling full, urge to eat, feeling low, level of tension, level of stress, level of anxiety) will be completed before and after the food tasks.
3. Computer tasks including the F-AAT [33] and the SRC [60] measuring approach bias towards high-calorie food items will be administered at baseline and after treatment.
4. Participants' body weight will be measured at baseline, after treatment and at follow-up.

#### Other clinical outcomes

1. Questionnaires assessing: depression - Depression, Anxiety and Stress Scale (DASS-21) [61], cognitive deficits - Montreal Cognitive Assessment (MoCA) [62], and impulsivity - Barratt Impulsiveness Scale (BIS-11) [63] will be administered at baseline and after treatment. The DASS-21 will also be administered at a two-week follow-up.
2. Symptoms of schizophrenia will be assessed by the Simplified Negative and Positive Symptoms Interview (SNAPSI) [64] and rated using the Positive and Negative Syndrome Scale (PANSS-6) [65] at baseline and after treatment.

#### Intervention-related outcomes

1. Acceptability of the intervention will be measured as follows: (a) before and after each treatment session by collecting VAS scores on the levels of tension, stress and anxiety; (b) before and after each treatment session by asking about any comments about the treatment; (c) at the end of the study, by asking participants if they would like to take part in a therapeutic trial of tDCS if this was available; and (d) by the number of recruited participants.
2. Treatment tolerability will be measured after each session by a VAS assessing levels of discomfort.

#### Blinding

This will be a double-blind study, where participants and researchers conducting assessments and delivering tDCS are blinded to treatment allocation. Sufficient blinding will be ensured by utilising a parallel design and a built-in neuroConn® DC-STIMULATOR blinding feature. With this, real and sham stimulations are assigned different codes, which the researcher enters into the device to start the stimulation. The real stimulation continues for 20 min, whereas the sham stimulation stops after 30 s, which triggers the same sensations on the skin (to improve blinding). To assess whether allocation concealment has been successful, participants and researchers will be asked to guess the treatment allocation at the end of the tDCS treatment and to indicate how certain they are of this guess. Participants will be debriefed and unblinded to group allocation at the end of the study. At that time, participants in the sham condition will be offered ABM + real tDCS treatment following the protocol as described above.

### Analyses

#### Feasibility

The decision as to whether to progress the study to a future large-scale RCT will be based on a number of criteria. These include the number of patients we are able to recruit, the proportion of patients retained, the proportion of patients completing the real and sham ABM + tDCS intervention, the acceptability and tolerability of the tDCS and the effect sizes of treatment outcomes. At the end of the study, these factors will be used to decide the case for progressing to a substantive RCT.

#### Clinical outcomes

Analyses will use the intention-to-treat principle. Descriptive statistical analyses and graphical methods will be used to determine quality, completeness and variability of the outcome measures. The size of the treatment effect on each outcome measure (questionnaires, tasks) will be the difference in outcome data between those in the two treatment conditions after treatment and at follow-up. Group differences will be estimated using linear mixed effects regression models, controlling for the baseline level of the outcome. The aim of the analysis is to establish a suitably precise effect size for the primary outcome at the post-treatment assessment in a future large-scale RCT.

## Discussion

Antipsychotic drug-induced weight gain can affect the physical health of people with schizophrenia. Additionally, it can cause individuals to discontinue medication and hence predispose them to relapse [1]. Interventions to prevent weight gain have limited effectiveness in acutely unwell patients who may find it hard to engage in diet or exercise behaviours [7]. There is a need for treatments to prevent weight gain that are easily accessible and can be utilised in various settings.

Non-invasive brain stimulation methods, e.g. tDCS, can be used to target brain areas such as the dlPFC which are associated with cognitive control (including eating) [24]. These have the potential to reduce antipsychotic drug-induced weight gain, e.g. by decreasing food cravings. TDCS can be combined with ABM training to strengthen its effects on retraining approach bias towards high calorie foods. We have described the protocol for a feasibility trial that will inform future studies and add to the evidence of brain-directed interventions for antipsychotic drug-induced weight gain.

Strengths of the study include use of combined neuromodulation and cognitive training. It has been reported that behaviours and cognitions undertaken during or following the tDCS can impair or abolish the effects of stimulation [66]. Administering two interventions concurrently will remove any cognitive interferences and ensure uniform treatment. The protocol is also designed to measure the possible mechanisms of action, e.g. on approach bias and impulsiveness, as well as on clinical symptoms. The protocol adheres to guidance on the optimal conduct of neuromodulation trials [55, 56, 67].

Possible challenges relate to recruitment/attrition. People with schizophrenia may be ambivalent about receiving tDCS because they may associate it with electroconvulsive therapy. Additionally, people who experience persecutory delusions may not want to undergo the treatment. As participants will be recruited via clinical teams, the aforementioned beliefs may also apply to the clinicians (i.e. the perceived value and cost of the treatment) and this may affect recruitment. To mitigate this, special care will be taken to explain the practical and technical nature of the tDCS to both service users and clinicians. Previous neuromodulation studies in patients with schizophrenia have showed good recruitment rates and adherence to treatment; however, it is unclear whether this can be replicated in the context of weight management. There may be other challenges, e.g. if the participant believes they are receiving sham, or if the treatment is too uncomfortable or too tiring. It is not clear whether participants will be able to distinguish between the real and sham treatment and what impact this will have on attrition rates. TDCS blinding is generally good, e.g. in an RCT for major depressive disorder, tDCS blinding was comparable to that of sertraline [68]. Sensations felt during treatment may interfere with blinding, tDCS can occasionally result in mild discomfort during administration (i.e. tingling, itching or skin redness). However, based on the review of 209 studies, these side effects occur at a similar rate in both real and sham groups [54].

In summary, combined tDCS and ABM is a promising brain-directed treatment for reducing food cravings and food consumption. This innovative feasibility RCT will assess the acceptability and efficacy of this intervention in people with schizophrenia. It will provide a basis for development of future large-scale RCTs and, if results are positive, will provide support for the implementation of it as a treatment.

## Trial status

Participant recruitment and data collection for this study began in June 2019. Recruitment will be completed in May 2020 (approximately). The most recent version of the protocol (v1.0 dated 4 April 2019) was approved by the Oxford B REC (reference no. 19/SW/0095) on 4 June 2019. Any substantial protocol amendments will be communicated to investigators via email and to other parties as required. Amendments to the study protocol will be reported in publications reporting the study outcomes.

## Supplementary information

**Additional file 1.** Populated SPIRIT checklist, consistent with the SPIRIT Statement (2013) [55] recommendations for reporting protocols.

## Data Availability

Not applicable.

## Abbreviations

5HT2a: 5-hydroxy-tryptamine receptor 2a
5HT2c: 5-hydroxy-tryptamine receptor 2c
ABM: Approach bias modification training
BIS-11: Barratt Impulsiveness Scale
BRC: Biomedical Research Centre
CONSORT: Consolidated Standards of Reporting Trials
D2: Dopamine receptor D2
DASS-21: Depression, Anxiety and Stress Scale
dlPFC: Dorsolateral prefrontal cortex
DSM-V: Diagnostic and Statistical Manual for Mental Disorders, Fifth edition
EDE-Q: Eating disorder examination questionnaire
F-AAT: Food Approach/Avoidance Task
FCQ-T-r: Food cravings questionnaire-trait-reduced
FCT: Food challenge task
fMRI: Functional magnetic resonance imaging
H1: Histamine receptor H1
H2: Histamine receptor H2
IoPPN: Institute of Psychiatry, Psychology and Neuroscience
ISRCTN: International Standard Randomised Controlled Trial Number
MC4R4: Melanocortin 4 receptor gene
MoCA: Montreal cognitive assessment
NIHR: National Institute for Health Research
PANSS-6: Positive and Negative Syndrome Scale
RCT: Randomised controlled trial
REC: Research ethics committee
rTMS: Repetitive transcranial magnetic stimulation
SLaM: South London and Maudsley NHS Foundation Trust
SNAPSI: Simplified Negative and Positive Symptoms Interview
SPIRIT: Standard Protocol Items Recommendations for Interventional Trials
SRC: Stimulus response compatibility task
tDCS: Transcranial direct current stimulation
VAS: Visual analogue scale
